# Transcranial Direct Current Stimulation (tDCS) over the Intraparietal Sulcus Does Not Influence Working Memory Performance

**DOI:** 10.5334/pb.534

**Published:** 2021-07-09

**Authors:** Romain Dumont, Steve Majerus, Michel Hansenne

**Affiliations:** 1 Psychology & Neuroscience of Cognition Research Unit, University of Liège, Belgium; 2 GIGA-CRC in vivo imaging Research Unit, University of Liège, Belgium; 3 Fund for Scientific Research FNRS, Belgium

**Keywords:** tDCS, Working memory, Intraparietal sulcus, Bayesian

## Abstract

Mixed results of the impact of transcranial direct current stimulation (tDCS) on working memory have been reported. Contrarily to previous studies who focused mainly on stimulating the dorsolateral prefrontal cortex, we modulated the left intraparietal sulcus (IPS) area which is considered to support attentional control aspects of working memory. Using a within-participant experimental design, participants completed three different conditions: anodal stimulation of the IPS, cathodal stimulation of the IPS, and sham stimulation of the IPS. Both visual and verbal working memory tasks were administered. In the visual task, participants had to memorize a random set of colored figures. In the verbal task, participants had to memorize a string of letters. Working memory load was manipulated in both tasks (six figures/letters vs. two figures/letters). No significant differences in accuracy or reaction time between the anodal, cathodal and sham conditions were found. Bayesian analysis supported evidence for an absence of effect. The results of the present study add to the growing body of contradictory evidence regarding the modulatory effects of single session tDCS on working memory performance.

## Introduction

Working memory (WM) involves a number of cognitive processes that allow us to keep active a limited amount of information for a brief period of time. It is a crucial ability for decision-making and reasoning (Diamond, 2013). WM capacity starts decreasing relatively early into adulthood (Park et al, 2002) and is frequently impaired after brain injury (Dunning, Westage, & Adam, 2016), in the context of developmental and psychiatric disorders (Kofler et al, 2018; Barch & Ceaser, 2012) or as a result of stress (Kim, Woo, & Woo, 2017).

Many studies have therefore tried to determine if WM capacity could be trained or enhanced, with highly variable degrees of success as regards both behavioral and neural stimulation techniques (Melby-Lervag & Hulme, 2013; Soveri et al, 2016). One of the neural stimulation techniques that is commonly used is transcranial direct current stimulation (tDCS). tDCS consists in the application of two sponge electrodes soaked in saline to the head through which a weak current (0.5–2 mA) is passed. After a few minutes of stimulation, the spontaneous neuronal activity in the cortex areas underlying the stimulations zones is either enhanced (anodal stimulation) or inhibited (cathodal stimulation) (Nitsche & Paulus, 2000). Anodal stimulation has mostly been used to increase behavioral performance while cathodal stimulation has been used to impair it. Stimulation effects have been shown to persist for a brief time even after stimulation has stopped (Kuo et al, 2013).

### tDCS and WM

Over the last fifteen years, a lot of studies have tried to use tDCS to improve WM. A number of these studies reported positive effects. A first study by Marshall, Mölle, Siebner, and Born (2005) applied anodal and cathodal tDCS bilaterally on the dorsolateral prefrontal cortex (DLPFC) while the participants performed a modified Sternberg task. The stimulation was performed intermittently (15 sec on/15 sec off) for 15 minutes at a low current strength of 260 µA. The authors found an increase in reaction time for both anodal and cathodal stimulation compared to sham, suggesting that tDCS influences neural processing of response selection and preparation. Soon after, Fregni et al. (2005) showed that anodal stimulation of the left DLPFC during a three-back task led to an increase in performance in terms of accuracy compared to sham stimulation while cathodal stimulation of the DLPFC and anodal stimulation of the primary motor cortex showed no differences.

Since then, some studies replicated these findings (Katsoulaki, Kastrinis, & Tsekoura, 2017 for a review) while others failed to report any significant effects of tDCS on WM performance (Robison, McGuirk, & Unsworth, 2017; Wang, Wen, & Li, 2018). More particularly, in a study consisting of four training sessions coupled with anodal tDCS over the DLPFC, Ruf, Fallgatter, and Plewnia (2017) showed significant improvement of WM performance on an N-back task. Moreover, this improvement appeared to be transferable to similar, untrained tasks, and to be long-lasting since an effect could still be objectified nine months after the training. Similar results have also been obtained by Au and colleagues (2016) showing that tDCS-related gains lasted for several months after seven training sessions. In addition, Bogdanov and Schwabe (2016) showed that anodal tDCS over the DLPFC could also significantly reduce the disruptive effect of stress on WM, and Brunyé, Moran, Holmes, Mahoney, and Taylor (2017) showed that anodal tDCS of the right fusiform gyrus could increase WM performance for face recognition but not for non-face objects (houses). On the other hand, quite a few studies revealed no significant effect of tDCS on WM. For instance, Nikolin, Martin, Loo, and Boonstra (2018) compared five groups of twenty subjects stimulated at five different intensities (2 mA, 1 mA, 0.034 mA, 0.016 mA and 0 mA) on the DLPFC and found no differences in performance between the groups in a visual 3-back WM task. Similarly, Robison and colleagues (2017) reported that stimulation of the prefrontal cortex and the posterior parietal cortex did not induce any significant differences in WM performance. Likewise, Nilsson, Lebedev, Rydström, and Lövdén (2017) did not observe any significant differences after twenty sessions of behavioral WM training coupled with anodal tDCS of the DLPFC.

Overall, most recent meta-analyses indicate the absence of reliable evidence for tDCS-related improvement of WM (Horvath, Forte, & Carter, 2015; Medina & Cason, 2017) or they indicate only very small effects (Hill, Fitzgerald, & Hoy, 2016; Mancuso, Ilieva, Hamilton, & Farah, 2016). Horvath and colleagues (2015) pooled together all cognitive outcome measures published in the literature and studied in healthy adult populations by at least two different research groups. They conducted 59 different analyses and not a single one of those showed a significant effect of the tDCS stimulation. Mancuso and colleagues (2016) focused their meta-analysis on studies assessing the effects of anodal stimulation of the right and left DLPFC and the right parietal lobe, as well as of the left DLPFC coupled with WM training. The only significant (albeit small) result reported by their meta-analysis was an improvement of WM performance following the left DLPFC stimulation coupled with WM training. Hill and colleagues (2016) investigated the effects of anodal tDCS on a variety of WM tasks and found a small but significant effect on reaction time (as well as a trend on accuracy) for offline tDCS. No effects were found for online tDCS. Medina and Cason (2017) used a meta-analytic tool developed by Simonsohn and colleagues (2014) for examining the distribution of published p-values as a function of expected distributions according to different effect sizes in the studies that Mancuso and colleagues (2016) had examined. By conducting this p-curve analysis they found no evidence that tDCS studies were associated with meaningful and significant effects.

### Alternative approaches of tDCS on WM

Two conclusions can be drawn in the light of the literature reviewed so far: Either the effects of tDCS on WM are very small (or non-existent), and therefore difficult to obtain and replicate, or tDCS has an effect but it depends on specific stimulation and task parameters that are currently unknown. The aim of the present study was to examine the second possibility, by using a theoretically and empirically informed stimulation approach of WM, based on recent developments in our knowledge about the cognitive and neural processes supporting both verbal and visuo-spatial WM.

Current theories of WM are placing strong emphasis on the role played by attention. In his integrated framework of WM and attention, Cowan (1995) considers that an essential component of WM is the focus and control of attention; these two components allow to hold memory content in an active state and are considered to define, at least partly, WM capacity. A number of other WM models have been described lending a central role to attention in WM performance (Barrouillet, Bernardin, & Camos, 2004; Kane & Engle, 2004).

The role of attentional processes in WM is supported by empirical evidence such as the involvement of two well-known attentional networks when participants perform WM tasks. Attention depends on at least two antagonistic networks in the brain, the dorsal attention network and the ventral attention network (Corbetta & Shulman, 2002; Vossel, Geng, & Fink, 2014). The dorsal attention network controls the voluntary, top-down deployment of attention to objects or locations, while the ventral attention network controls the reorientation of attention to sensory stimulation provoked by unattended or unexpected stimuli (bottom-up attention). The dorsal network comprises the intraparietal sulcus (IPS) and the frontal eye fields (FEF) of each hemisphere, whereas the temporoparietal junction (TPJ) and the ventral frontal cortex (VFC) are the main regions of the ventral network. These two networks have been shown to intervene also in WM tasks, and this in a load dependent and antagonistic manner, for both verbal and visual WM tasks. The dorsal attention network shows increased activity with increasing WM load while the ventral attention network shows WM load-dependent decreases of activity (Majerus et al., 2010, 2012, 2016; Todd & Marois, 2004; Todd, Fougnie, & Marois., 2005; Xu & Chun, 2006).

Given the central role of the dorsal attention network centered on the left IPS in WM tasks, the present tDCS study focused on the modulatory effects of left IPS stimulation on verbal and visual WM performance. Some previous tDCS studies have already focused on the stimulation of the IPS and have reported positive results, although not directly in a WM context. For instance, the bilateral stimulation of the IPS induced better performance on mental addition but not on a Stroop task, (Klein et al., 2013; Artemenko, Moeller, Huber, & Klein, 2015), whereas stimulation over the left IPS induced better subsequent recognition memory in verbal episodic memory tasks (Jacobson, Goren, Lavidor, & Levy, 2012). It has been demonstrated also that anodal stimulation over the right IPS improved object tracking performance in a high load condition but not in a low load condition, which is consistent with the memory load effect on the IPS activity (Blumberg, Peterson, & Parasuraman, 2015). In addition, stimulation over the right IPS affected performance in a visual attention task (Moos, Vossel, Weidner, Sparing, & Fink, 2012). However, a recent tDCS study by Nikolin, Lauf, Loo, and Martin (2019) showed no significant effect after left IPS stimulation on an n-back WM task. It should however be noted that they observed moderate effect sizes on WM tasks (e.g., 0.38 for the 3-back task), and the authors argued that the lack of significance could have been the result of the high inter-individual variability in performance.

The aim of the present study is to extend these emerging findings by determining whether it is possible to increase and also decrease performance in a WM task through modulation of IPS activity associated with the dorsal attention network. Consistently with previous results, we hypothesize that anodal stimulation of the left IPS would enhances the dorsal attention network and leads to increase of WM performance but only in the high WM load condition. Conversely, we assume that cathodal stimulation of the left IPS would inhibit the dorsal attention network and lead to decreased WM performance for the high WM load condition. tDCS effects are not expected in the low WM load because this condition is associated with high success rate and does not push WM capacity to its limit. We will use Bayesian statistics to quantify the evidence both for and against the existence of an effect.

## Methods

### Participants

Using G*Power 3.1 (Faul, Erdfelder, Buchner, & Lang, 2009) we determined that to achieve a power of 0.8, an α-error of 0.05 and an estimated effect size of 0.4, we would require at least 42 participants. Fifty right-handed participants were recruited from the university community to take part in the experiment. Forty-seven participants (twenty-three male; mean age = 24.15, SD = 2.05) were retained for the final analysis; three participants had to be excluded due to problems in data collection. More precisely, two participants had no recorded data in one of their sessions and the third had a very low response rate across all sessions (more than 80% abstention rate). Participants were free of any history of psychiatric or neurological diseases and from any form of colorblindness. They gave their written informed consent to take part in the study, which was approved by the Ethics Committee of the Psychology Faculty of the Liège University, Belgium. The participants did not receive any compensation for their participation.

### General Procedure

All participants took part in three separate tDCS sessions in a pseudo-randomized order approximately one week (±2 days) apart. In each session, one of the three conditions, anodal stimulation, cathodal stimulation or sham stimulation, was administered. The participants completed two WM tasks (adapted from Majerus et al., 2016) during each of three sessions, a visual array probe recognition task and a letter probe recognition task. These tasks were chosen because they have been shown to recruit the dorsal attention network and more specifically the bilateral IPS, and this particularly for higher WM load (Majerus et al., 2016): both tasks lead to increased neural activity and differential multivariate voxel patterns in the IPS for 6-load versus 2-load conditions. The tasks were explained verbally to the participants and they had the opportunity to ask further questions about the task. They also conducted a short practice session for each task (5 trials).

### WM tasks

**Figure 1:**
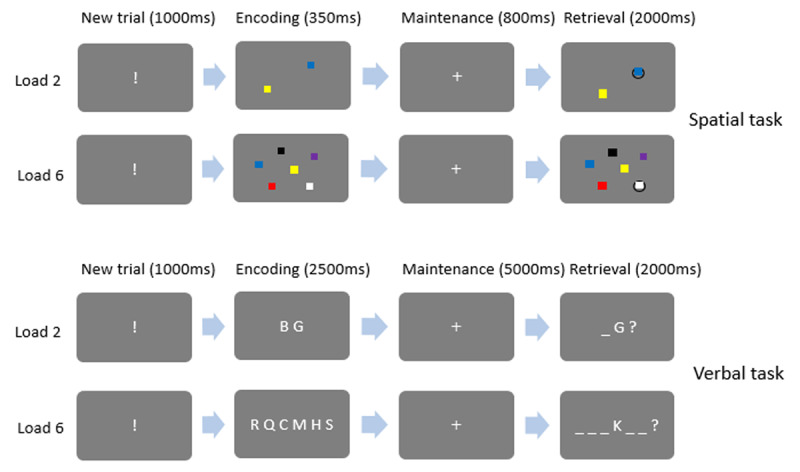
Schematic drawing of the spatial and verbal WM tasks.

In the visuo-spatial WM task (visual array probe recognition; Luck & Vogel, 1997), the participants had to memorize the position of multiple colored squares (see ***Figure 1***). They were comfortably seated at a distance of 90 centimeters from a 22-inch screen. Each trial started with the presentation of an exclamation mark during 1000 ms. Next, an array containing 2 or 6 colored squares appeared on a grey background. The squares disappeared after 350 ms, but the grey background stayed for another 800 ms. After that, the array reappeared on the screen with one of the squares circled and the participants had to decide in less than 2000 ms whether the circled square was of the same color as in the memory array or not. If the color was different, it was also different from the color of the other squares for this trial. For a given trial, no two squares were ever of the same color. Thus, if the square being probed was of a different color than the target square, the new color was also different from the color of any other square, thereby discarding the possibility that negative probes could be rejected merely by detecting color similarity between squares of the probe array. The participants answered by pressing one of two response keys on the computer keyboard (‘d’ with their left index for ‘same color’ and ‘k’ with their right index for ‘different color’). Once an answer had been recorded or the time limit of 2000 ms had been reached there was an inter-trial interval of variable duration (random Gaussian distribution centered on a mean duration of 4000 ± 1000 ms) before the next trial was initiated. This task was constructed to capture non-strategic, attention-based maintenance mechanisms via brief presentation and maintenance durations. There was a total of 60 trials with half of them featuring a color change. Half of the trials comprised 2 squares (low load condition) and half comprised 6 squares (high load condition). Final scores were standardized to obtain a percentage of correct, incorrect and missing responses and their corresponding average reaction times.

In the verbal WM task (letter probe recognition task, Sternberg, 1966), the participants were presented horizontal sequences of 2 or 6 letters, the letters being sampled without repetition from a pool of 16 different consonants (see ***Figure 1***). The presentation apparatus was the same as for the visual WM task. Each trial started with the presentation of an exclamation mark for 1000 ms. The memory sequence then appeared for 2500 ms followed by a fixation cross for 4000 ms during which the participants had to maintain the sequence in memory. A probe letter then appeared in one of the 2 or 6 possible serial positions, the serial positions being indicated by a sequence of horizontal bars. The participants had 2000 ms to decide if the probe letter had been in the memory list and if it had appeared in the same position as in the probe array. The participants answered by pressing one of two response keys on the computer keyboard (‘d’ with their left index for ‘correct’ and ‘k’ with their right index for ‘incorrect’). Once an answer had been recorded or the time limit of 2000 ms had been reached there was an inter-trial interval of variable duration (random Gaussian distribution centered on a mean duration of 4000 ± 1000 ms) before the next trial was initiated. It should be noted that there were trials where the letter was present but on another position, but there were no trials where the probe letter was part of the previous list. There was a total of 84 trials, with the same number of trials for each load condition; half of the probes were matching probes. Final scores were standardized to obtain a percentage of correct, incorrect and missing responses and their corresponding average reaction times.

### tDCS

The stimulation was delivered by The Brain Stimulator 1 system (The Brain Stimulator Inc., San Jose, California, USA) through a pair of saline-soaked surface sponge electrodes (target electrode = 3 × 3 cm, return electrode = 5 × 5 cm) connected to a battery-driven constant current stimulator. For the anodal condition, the anode electrode was placed over the left IPS (P3 localization according to the 10/20 EEG international system), and the cathode was placed on the right cheek, and for the cathodal condition, the cathode was placed over the left IPS and the anode on the right cheek. Active stimulation consisted of a constant current of 2 mA applied for 20 min (with 30 seconds of fade-in and fade-out), corresponding to the duration of the task. The sham condition consisted of stimulation for 30s, and then the stimulator was turned off. After the stimulation participants were asked if they felt strong discomfort regarding the stimulation and if they felt any side effects of the stimulation. Two participants complained about slight dizziness following anodal stimulation and another one following cathodal stimulation. Note however that there was no more formal assessment of side effects.

### Statistical analyses

A 3 (anodal, cathodal vs sham condition) × 2 (low vs high load) repeated-measures analysis of variance (ANOVA) was conducted on both response accuracy and reaction times for each task (visuo-spatial and verbal) with JASP (JASP Team, 2019, version 0.9.2). The Shapiro-Wilk test was applied to assess the normality of the data and Mauchly’s test was used to assess the sphericity assumption. Greenhouse-Geisser-corrected significance values were used when the sphericity assumption was not met.

Furthermore, we conducted Bayesian analyses to assess evidence for the null hypothesis. Bayesian analyses were conducted with JASP (version 0.9.2) with default prior settings (all models have equal prior probabilities). Since there was a total of five models all prior probabilities were set to 0.2. The null model includes no predictor variable. Contrary to frequentist statistics, the obtained Bayes factors estimate evidence in favor of either the null hypothesis (BF_01_) or the effect of interest (BF_10_). We used Jeffreys’ indicative benchmarks (1998) to describe the strength of evidence as anecdotal (0–3), substantial (3–10), strong (10–30), very strong (30–100) or decisive (>100).

## Results

**Figure 2:**
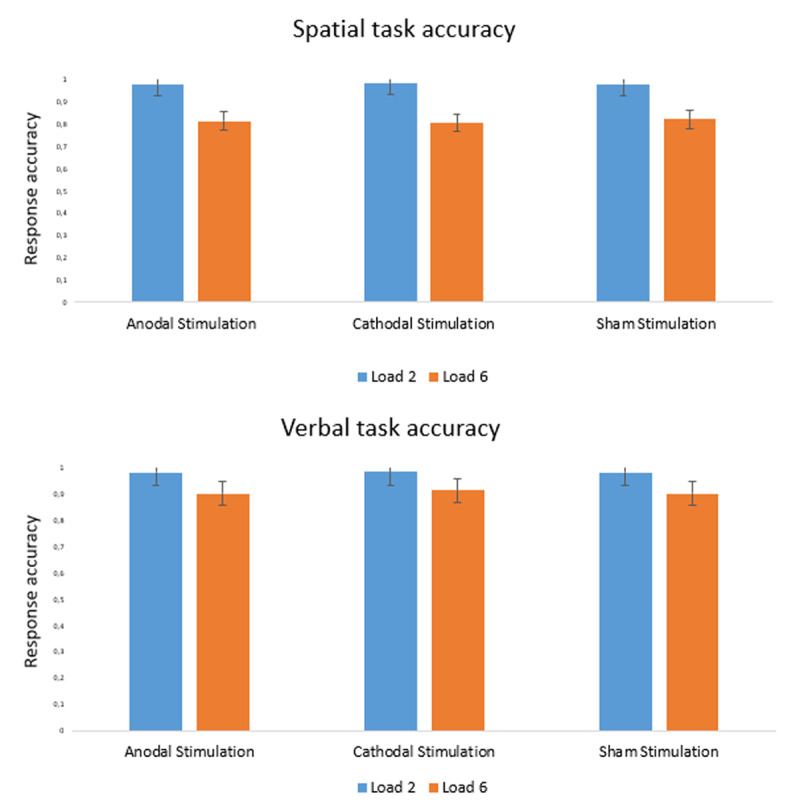
Accuracy results of the visuo-spatial and verbal tasks.

A first repeated-measures analysis of variance (ANOVA) was conducted on response accuracy for both tasks (see ***Figure 2***). The percentages of accuracy for the visuo-spatial task were 97.8% (load 2) and 81.5% (load 6) in the anodal stimulation condition, 98,3% (load 2) and 81.5% (load 6) in the cathodal stimulation condition, and 98,3% (load 2) and 80.7% (load 6) in the sham condition, respectively. For the verbal task the percentages of accuracy were 98.3% (load 2) and 90.4% (load 6) in the anodal stimulation condition, 98.6% (load 2) and 91.6% (load 6)) in the cathodal stimulation condition, and 98.4% (load 2) and 90.3% (load 6) in the sham condition, respectively. The ANOVAs showed a significant main effect of load on performance (visuo-spatial: F(1,46) = 277.42, p < 0.001, η_p_^2^ = 0.86; verbal: F(1,46) = 63.40, p < 0.001, η_p_^2^ = 0.58), but no main effect of condition (visuo-spatial: F(2,92) = 0.29, p = 0.75; verbal: F(2,92) = 1.27, p = 0.29) nor a significant condition × load interaction (visuo-spatial: F(2,92) = 1.02, p = 0.37; verbal: F(2,92) = 0.76, p = 0.47). These results were confirmed by Bayesian analysis, the model associated with the strongest evidence including only the effect of load (visuo-spatial: BF_10_ = 4.91e+62; verbal: BF_10_ = 1.90e+20); strong evidence *against* a condition effect was observed (visuo-spatial: BF_01_ = 24.33; verbal: BF_01_ = 24.92).

**Figure 3:**
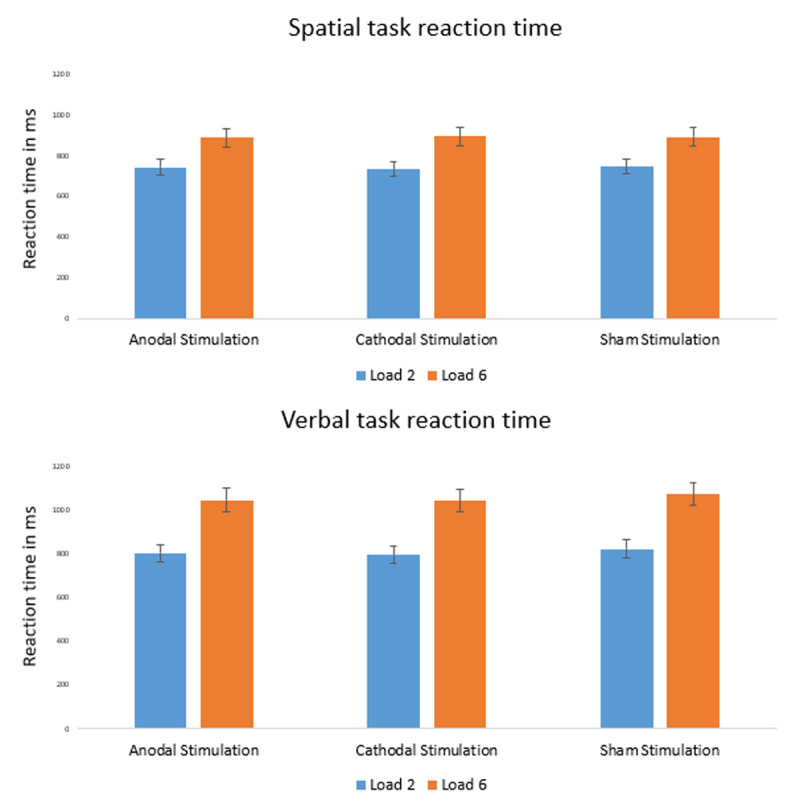
Reaction time results of the visuo-spatial and verbal tasks.

Next, we ran the same ANOVA on reaction times (see ***Figure 3***). The results showed again a significant effect of load (visuo-spatial: F(1,46) = 193.69, p < 0.001, η_p_^2^ = 0.81; verbal: F(1,46) = 646.39, p < 0.001, η_p_^2^ = 0.93), but no main effect of condition (visuo-spatial: F(2,92) = 0.03, p = 0.97; verbal: F(2,92) = 1.37, p = 0.26) nor any significant condition × load interaction (visuo-spatial: F(2,92) = 0.76, p = 0.47; verbal: F(2,92) = 0.21, p = 0.82). These results were again confirmed by Bayesian analysis, the model associated with the strongest evidence only including the main effect of load (visuo-spatial: BF_10_ = 1.94e+27; verbal: BF_10_ = 2.68e+57); strong evidence *against* a condition effect was observed (visuo-spatial: BF_01_ = 17.29; verbal: BF_01_ = 13.13).

Lastly, in an effort to rule out any adverse effect of training between sessions we performed separate analyses for each session, we restricted the analyses to response accuracy. In the main analyses, there was only one group of subjects while in the following analyses we will assess the effect of tDCS condition for each session individually implying that the tDCS condition effect will become a group effect (a given participant can only receive one stimulation condition for a given session).

### Session 1

A repeated-measures analysis of variance (ANOVA) was conducted on response accuracy for both tasks during the first tDCS session. The results showed a significant main effect of load (visuo-spatial: F(1,44) = 227.34, p < 0.001, η_p_^2^ = 0.84; verbal: F(1,44) = 65.80, p < 0.001, η_p_^2^ = 0.60), but no main effect of condition (visuo-spatial: F(2,44) = 0.97, p = 0.389; verbal: F(2,44) = 1.19, p = 0.332) nor a significant condition × load interaction (visuo-spatial: F(2,44) = 0.15, p = 0.865; verbal: F(2,44) = 1.13, p = 0.332). These results were confirmed by Bayesian analysis, the model associated with the strongest evidence including only the effect of load (visuo-spatial: BF_10_ = 9.39e+21; verbal: BF_10_ = 8.76e+9); the condition variable was associated with substantial evidence *against* its effect (visuo-spatial: BF_01_ = 6.42; verbal: BF_01_ = 4.89).

### Session 2

When running the same analysis for response accuracy in Session 2, we observed again a significant main effect of load (visuo-spatial: F(1,44) = 148.04, p < 0.001, η_p_^2^ = 0.77; verbal: F(1,44) = 53.90, p < 0.001, η_p_^2^ = 0.55), but no main effect of condition (visuo-spatial: F(2,44) = 1.13, p = 0.331; verbal: F(2,44) = 0.59, p = 0.559) nor a significant condition × load interaction (visuo-spatial: F(2,44) = 0.942, p = 0.397; verbal: F(2,44) = 0.91, p = 0.41). These results were confirmed by Bayesian analysis, the model associated with the strongest evidence including only the effect of load (visuo-spatial: BF_10_ = 2.13e+17; verbal: BF_10_ = 6.12e+8); the condition variable was associated with substantial evidence *against* its effect (visuo-spatial: BF_01_ = 5.77; verbal: BF_01_ = 6.29).

### Session 3

Finally, the same analyses were conducted for Session 3 and revealed again a significant main effect of load (visuo-spatial: F(1,44) = 151.73, p < 0.001, η_p_^2^ = 0.78; verbal: F(1,44) = 28.41, p < 0.001, η_p_^2^ = 0.39), but no main effect of condition (visuo-spatial: F(2,44) = 0.30, p = 0.739; verbal: F(2,44) = 0.74, p = 0.485) nor a significant condition × load interaction (visuo-spatial: F(2,44) = 0.30, p = 0.745; verbal: F(2,44) = 0.89, p = 0.417). Bayesian analysis confirmed that the model associated with the strongest evidence included only the effect of load (visuo-spatial: BF_10_ = 1.51e+19; verbal: BF_10_ = 5.47e+4); the condition variable was once more associated with substantial evidence *against* its effect (visuo-spatial: BF_01_ = 7.90; verbal: BF_01_ = 5.56).

### Linear mixed effects model

Finally, we conducted linear mixed effects analyses in order to better account for inter-individual variability and associated differences in participants’ intercepts. A first linear mixed effects model, with subjects as random variable, on response accuracy in the visuo-spatial task revealed no significant difference in performance as a function of condition (Χ^2^(2) = 0.542; p = 0.763) but still highlighted a significant effect of load (Χ^2^(1) = 561.545; p < 0.001). The same analysis on response accuracy in the verbal task also showed no significant difference in performance as a function of condition (Χ^2^(2) = 1.607; p = 0.448) but again a significant effect of load was observed (Χ^2^(1) = 173.833; p < 0.001). Finally, the same analysis on response times for the visuo-spatial task showed no significant condition effect (Χ^2^(2) = 0.119; p = 0.942) but a significant effect of load (Χ^2^(1) = 123.016; p < 0.001). The same results were observed for reactions times in the verbal task: no condition effect (Χ^2^(2) = 5.023; p = 0.081) but once more a significant load effect (Χ^2^(1) = 521.570; p < 0.001).

## Discussion

The aim of this study was to determine the impact of anodal and cathodal tDCS of the IPS on verbal and visuo-spatial WM performance. Because of the inconsistencies of the effect of tDCS stimulation of DLPFC on WM performance, we decided to target the IPS, a region specifically associated to attentional control components of WM. Since strong evidence supports the implication of the IPS in attentional components of WM (Majerus et al., 2016; Todd & Marois, 2004, Todd, Fougnie, & Marois., 2005), we hypothesized that anodal stimulation of the IPS would enhance dorsal attention network activity and lead to increased WM performance, and this specificallyin the high load condition. Conversely, we assumed that cathodal stimulation of the IPS would inhibit the dorsal attention network and lead to decreased WM performance for the high load condition. tDCS effects were not expected to occur in the low load condition because this condition is associated with high success rate and does not exceed WM capacity, and hence there is no room for a tDCS booster effect on cognitive performance (Roe et al., 2016). Despite that the study was based on a robust theoretical background and used standard stimulation parameters (i.e., 2 mA tDCS applied for 20 min during task performance), no effect of stimulation was found. Furthermore, thanks to Bayesian analysis we can make safe conclusions about the absence of an effect of tDCS in this study.

A few limitations to our study need be acknowledged. First, given the already important number of conditions there was neither a baseline condition nor an offline condition that could have helped quantify potential placebo effects and short-term effects of tDCS, respectively. In tDCS research, a baseline measure is a measure of a participant’s performance without any tDCS intervention. Indeed, sham stimulation can have an impact on participants through the placebo effect, or, alternatively, other factors like the stress or the discomfort caused by the device can have nocebo effects (see Fonteneau et al. (2019) for a review). Baseline measures allow to measure these biases and to determine more accurately a participant’s natural levels of performance, relative to the different tDCS conditions. An offline condition refers to the condition where WM performance is assessed after the participants have been stimulated by tDCS. This condition makes it possible to study long-term and distance effects of tDCS. For example, Friehs and Frings (2019) observed greater offline effects of tDCS compared to online effects. Another limitation of the study is that our tDCS device did not allow for impedance monitoring. However, the device comprised a current regulator ensuring constant current levels despite impedance variation.

A potential explanation for the absence of stimulation effects in the present study could be that despite careful positioning of the electrodes current flow partially or totally missed the target area (Chib et al., 2013 for example). Thair et al. (2017) list a large number of factors capable of influencing current flow (hair thickness, sweat, head size, skull thickness,…). This is a general limitation applying to tDCS technology. It may also be possible that the tDCS effect is too weak to increase or decrease WM performance in a young and healthy population of university students or that the dorsal attention network does not play a critical role in our task even if fMRI studies have shown that this network is consistently associated with high load WM tasks as used here. Alternatively, since WM is associated to a large fronto-parietal, bilateral network (Eriksson, Vogel, Lansner, Bergström, & Nyberg, 2015), the non-stimulated parts of this network (e.g., DLPFC, right IPS) could have exerted a compensatory effect to the stimulated, left IPS part of the network (Heekeren et al., 2008). This explanation is of course only valid for cathodal stimulation of the IPS. A further explanation for the absence of effect of anodal stimulation could be that baseline WM capacity of our participants was too high (all being young university students) and that there was little room for improvement. Indeed, Gözenman and Berryhill (2016) showed that a group displaying lower baseline WM capacity benefitted to a greater extent from tDCS than a group with a higher baseline WM capacity.

The results of the present study are not the first reporting null effect of tDCS on WM or on other cognitive abilities and are consistent with the meta-analytic studies that have highlighted the problem of mixed and contradictory findings about single session tDCS effects in young and healthy participants (Westwood and Romani, 2018; Lukasik et al., 2018; Meier and Sauter, 2018). In addition, some studies that have shown a beneficial role of tDCS stimulation over the DLPFC, nevertheless also report important individual differences with TDCS-related WM performance increase in some participants, and performance decrease or no effect in other participants (Talsma et al., 2017).

Before concluding to the absence of an effect or very limited effects of tDCS on WM, future studies must try to replicate previous positive findings by using the the same methodology as used in these original studies. Indeed, differences in stimulation protocols in terms of intensity and duration of stimulation, site of the stimulation, type of WM task and difficulty, and type of population (young healthy vs mid-aged or old participants) could partly account for the mixed results reported in the literature. Also, the use of a higher WM load (e.g., 8 items) would avoid potential ceiling effects and leave more room for tDCS-related improvement. For example, Roe et al. (2016) have shown tDCS effects in a visual working memory task only when the WM load exceeded the participants’ cognitive resources. This being said, the effect of WM load was nevertheless quite robust in the present study and the study by Majerus et al. (2016) had shown reliable univariate and multivariate neural changes in the IPS when comparing WM sets of 6 vs. 2 items. Adding both baseline and offline measurements as done in some studies with significant results (Friehs and Frings, 2019) would also allow to investigate effects of tDCS on WM performance more comprehensively.

As already noted, the impact of important individual differences in WM capacity also needs to be considered, as tDCS effects could be more pronounced in participants characterized by poor WM capacity, even if a previous study failed to observe such an effect in young participants (Westwood and Romari, 2018). It could also be useful to consider individual differences in encoding strategies as they have been shown to mediate the effect of tDCS on the posterior parietal cortex in a visual short-term memory task (Wang, Itthipuripat, & Ku, 2020). In that study, two groups of subjects took part in a visual working memory task using different encoding strategies. While the group focusing on remembering all stimuli was not affected by tDCS the group using a focused attention strategy, with attention oriented towards a specific subset of stimuli, showed significant effects of tDCS.

In conclusion, the results of the present study do not demonstrate an effect of tDCS on WM performance, in line with a number of studies that have shown mixed or negative results regarding WM performance modulation in young, healthy participants after a single tDCS session. Future experiments should be conducted to assess the effective neuromodulatory action of tDCS as well as the impact of differences in stimulation protocol and intensity.

## References

[1] Artemenko, C., Moeller, K., Huber, S., & Klein, E. (2015). Differential influences of unilateral tDCS over the intraparietal cortex on numerical cognition. Frontiers in Human Neuroscience, 9, 110. DOI: 10.3389/fnhum.2015.00110 25798099 PMC4350389

[2] Au, J., Katz, B., Buschkuehl, M., Bunarjo, K., Senger, T., Zabel, C., Jaeggi, S. M., & Jonides, J. (2016). Enhancing working memory training with transcranial direct current stimulation. Journal of Cognitive Neuroscience, 28, 1419–1432. DOI: 10.1162/jocn_a_00979 27167403

[3] Barch, D. M., & Ceaser, A. (2012). Cognition in schizophrenia: Core psychological and neural mechanisms. Trends in Cognitive Sciences, 16(1), 27–34. DOI: 10.1016/j.tics.2011.11.015 22169777 PMC3860986

[4] Barrouillet, P., Bernardin, S., & Camos, V. (2004). Time constraints and resource sharing in adults’ working memory spans. Journal of Experimental Psychology: General, 133, 83–100. DOI: 10.1037/0096-3445.133.1.83 14979753

[5] Blumberg, E. J., Peterson, M. S., & Parasuraman, R. (2015). Enhancing multiple object tracking performance with noninvasive brain stimulation: a causal role for the anterior intraparietal sulcus. Frontiers in Systems Neuroscience, 9, 3. DOI: 10.3389/fnsys.2015.00003 25698943 PMC4318277

[6] Bogdanov, M., & Schwabe, L. (2016). Transcranial Stimulation of the Dorsolateral Prefrontal Cortex Prevents Stress-Induced Working Memory Deficits. Journal of Neuroscience, 36, 1429–1437. DOI: 10.1523/JNEUROSCI.3687-15.2016 26818528 PMC6604824

[7] Brunyé, T. T., Moran, J. M., Holmes, A., Mahoney, C. R., & Taylor, H. A. (2017). Non-invasive brain stimulation targeting the right fusiform gyrus selectively increases working memory for faces. Brain and Cognition, 113, 32–39. DOI: 10.1016/j.bandc.2017.01.006 28107684

[8] Chib, V. S., Yun, K., Takahashi, H., & Shimojo, S. (2013). Noninvasive remote activation of the ventral midbrain by transcranial direct current stimulation of prefrontal cortex. Translational Psychiatry, 3, e268–e268. DOI: 10.1038/tp.2013.44 23756377 PMC3693403

[9] Corbetta, M., & Shulman, G. L. (2002). Control of goal-directed and stimulus-driven attention in the brain. Nature Reviews Neuroscience, 3, 201–215. DOI: 10.1038/nrn755 11994752

[10] Cowan, N. (1995). Attention and memory: An integrated framework. Oxford University Press. DOI: 10.1093/acprof:oso/9780195119107.001.0001

[11] Diamond, A. (2013). Executive functions. Annual Review of Psychology, 64, 135–168. DOI: 10.1146/annurev-psych-113011-143750 PMC4084861 23020641

[12] Dunning, D. L., Westgate, B., & Adlam, A. L. R. (2016). A meta-analysis of working memory impairments in survivors of moderate-to-severe traumatic brain injury. Neuropsychology, 30, 811–819. DOI: 10.1037/neu0000285 27182710

[13] Eriksson, J., Vogel, E. K., Lansner, A., Bergström F., & Nyberg, L. (2015). Neurocognitive architecture of working memory. Neuron, 88, 33–46. DOI: 10.1016/j.neuron.2015.09.020 26447571 PMC4605545

[14] Faul, F., Erdfelder, E., Buchner, A., & Lang, A. G. (2009). Statistical power analyses using G* Power 3.1: Tests for correlation and regression analyses. Behavior Research Methods, 41, 1149–1160. DOI: 10.3758/BRM.41.4.1149 19897823

[15] Fonteneau, C., Mondino, M., Arns, M., Baeken, C., Bikson, M., Brunoni, A. R., Burke, M. J., Neuvonen, T., Padberg, F., Pascual-Leone, A., Poulet, E., Ruffini, G., Santarnecchi, E., Sauvaget, A., Schellhorn, K., Suaud-Chagny, M. F., Palm, U., & Brunelin, J. (2019). Sham tDCS: A hidden source of variability? Reflections for further blinded, controlled trials. Brain Stimulation, 12, 668–673. DOI: 10.1016/j.brs.2018.12.977 30639235

[16] Fregni, F., Boggio, P. S., Nitsche, M., Bermpohl, F., Antal, A., Feredoes, E., … & Pascual-Leone, A. (2005). Anodal transcranial direct current stimulation of prefrontal cortex enhances working memory. Experimental Brain Research, 166, 23–30. DOI: 10.1007/s00221-005-2334-6 15999258

[17] Friehs, M. A., & Frings, C. (2019). Offline beats online: transcranial direct current stimulation timing influences on working memory. Neuroreport, 30, 795–799. DOI: 10.1097/WNR.0000000000001272 31283711

[18] Gözenman, F., & Berryhill, M. E. (2016). Working memory capacity differentially influences responses to tDCS and HD-tDCS in a retro-cue task. Neuroscience Letters, 629, 105–109. DOI: 10.1016/j.neulet.2016.06.056 27369325 PMC4983211

[19] Heekeren, H. R., Marrett, S., & Ungerleider, L. G. (2008). The neural systems that mediate human perceptual decision making. Nature Reviews Neuroscience, 9(6), 467–479. DOI: 10.1038/nrn2374 18464792

[20] Hill, A. T., Fitzgerald, P. B., & Hoy, K. E. (2016). Effects of anodal transcranial direct current stimulation on working memory: a systematic review and meta-analysis of findings from healthy and neuropsychiatric populations. Brain Stimulation, 9, 197–208. DOI: 10.1016/j.brs.2015.10.006 26597929

[21] Horvath, J. C., Forte, J. D., & Carter, O. (2015). Quantitative review finds no evidence of cognitive effects in healthy populations from single-session transcranial direct current stimulation (tDCS). Brain Stimulation, 8, 535–550. DOI: 10.1016/j.brs.2015.01.400 25701175

[22] Jacobson, L., Goren, N., Lavidor, M., & Levy, D. A. (2012). Oppositional transcranial direct current stimulation (tDCS) of parietal substrates of attention during encoding modulates episodic memory. Brain Research, 1439, 66–72. DOI: 10.1016/j.brainres.2011.12.036 22265704

[23] JASP Team. (2019). JASP(Version 0.9.2) [Computer software] (BibTeX).

[24] Jeffreys, H. (1998). The theory of probability. OUP Oxford.

[25] Katsoulaki, M., Kastrinis, A., & Tsekoura, M. (2017). The effects of anodal transcranial direct current stimulation on working memory. GeNeDis 2016, 283–289. DOI: 10.1007/978-3-319-57379-3_25 28971466

[26] Kim, Y., Woo, J., & Woo, M. (2017). Effects of Stress and Task Difficulty on Working Memory and Cortical Networking. Perceptual and Motor Skills, 124, 1194–1210. DOI: 10.1177/0031512517732851 28942702

[27] Kofler, M. J., Sarver, D. E., Harmon, S. L., Moltisanti, A., Aduen, P. A., Soto, E. F., & Ferretti, N. (2018). Working memory and organizational skills problems in ADHD. Journal of Child Psychology and Psychiatry, 59, 57–67. DOI: 10.1111/jcpp.12773 28714075 PMC5729117

[28] Kuo, H. I., Bikson, M., Datta, A., Minhas, P., Paulus, W., Kuo, M. F., & Nitsche, M. A. (2013). Comparing cortical plasticity induced by conventional and high-definition 4 × 1 ring tDCS: a neurophysiological study. Brain Stimulation, 6, 644–648. DOI: 10.1016/j.brs.2012.09.010 23149292

[29] Luck, S. J., & Vogel, E. K. (1997). The capacity of visual working memory for features and conjunctions. Nature, 390, 279–281. DOI: 10.1038/36846 9384378

[30] Lukasik, K. M., Lehtonen, M., Salmi, J., Meinzer, M., Joutsa, J., & Laine, M. (2018). No effects of stimulating the left ventrolateral prefrontal cortex with tDCS on verbal working memory updating. Frontiers in Neuroscience, 11, 738. DOI: 10.3389/fnins.2017.00738 29379410 PMC5770813

[31] Majerus, S., Attout, L., D’Argembeau, A., Degueldre, C., Fias, W., Maquet, P., … & Balteau, E. (2012). Attention supports verbal short-term memory via competition between dorsal and ventral attention networks. Cerebral Cortex, 22, 1086–1097. DOI: 10.1093/cercor/bhr174 21765184

[32] Majerus, S., Cowan, N., Péters, F., Van Calster, L., Phillips, C., & Schrouff, J. (2016). Cross-modal decoding of neural patterns associated with working memory: Evidence for attention-based accounts of working memory. Cerebral Cortex, 26, 166–179. DOI: 10.1093/cercor/bhu189 25146374 PMC4717284

[33] Majerus, S., D’Argembeau, A., Martinez Perez, T., Belayachi, S., Van der Linden, M., Collette, F., … & Maquet, P. (2010). The commonality of neural networks for verbal and visual short-term memory. Journal of cognitive neuroscience, 22, 2570–2593. DOI: 10.1162/jocn.2009.21378 19925207

[34] Mancuso, L. E., Ilieva, I. P., Hamilton, R. H., & Farah, M. J. (2016). Does transcranial direct current stimulation improve healthy working memory?: a meta-analytic review. Journal of Cognitive Neuroscience, 28, 1063–1089. DOI: 10.1162/jocn_a_00956 27054400

[35] Marshall, L., Mölle, M., Siebner, H. R., & Born, J. (2005). Bifrontal transcranial direct current stimulation slows reaction time in a working memory task. BMC Neuroscience, 6, 1–7. DOI: 10.1186/1471-2202-6-23 15819988 PMC1090588

[36] Medina, J., & Cason, S. (2017). No evidential value in samples of transcranial direct current stimulation (tDCS) studies of cognition and working memory in healthy populations. Cortex, 94, 131–141. DOI: 10.1016/j.cortex.2017.06.021 28759803

[37] Meier, B., & Sauter, P. (2018). Boosting memory by tDCS to frontal or parietal brain regions? A study of the enactment effect shows no effects for immediate and delayed recognition. Frontiers in Psychology, 9, 867. DOI: 10.3389/fpsyg.2018.00867 29915551 PMC5994422

[38] Melby-Lervåg, M., & Hulme, C. (2013). Is working memory training effective? A meta-analytic review. Developmental Psychology, 49, 270–291. DOI: 10.1037/a0028228 22612437

[39] Moos, K., Vossel, S., Weidner, R., Sparing, R., & Fink, G. R. (2012). Modulation of top-down control of visual attention by cathodal tDCS over right IPS. Journal of Neuroscience, 32, 16360–16368. DOI: 10.1523/JNEUROSCI.6233-11.2012 23152618 PMC6794038

[40] Nikolin, S., Lauf, S., Loo, C. K., & Martin, D. (2019). Effects of high-definition transcranial direct current stimulation (HD-tDCS) of the intraparietal sulcus and dorsolateral prefrontal cortex on working memory and divided attention. Frontiers in Integrative Neuroscience, 12, 64. DOI: 10.3389/fnint.2018.00064 30670954 PMC6331442

[41] Nikolin, S., Martin, D., Loo, C. K., & Boonstra, T. W. (2018). Effects of TDCS dosage on working memory in healthy participants. Brain Stimulation, 11, 518–527. DOI: 10.1016/j.brs.2018.01.003 29361442

[42] Nilsson, J., Lebedev, A. V., Rydström, A., & Lövdén, M. (2017). Direct-current stimulation does little to improve the outcome of working memory training in older adults. Psychological Science, 28, 907–920. DOI: 10.1177/0956797617698139 28509625 PMC5536199

[43] Nitsche, M. A., & Paulus, W. (2000). Excitability changes induced in the human motor cortex by weak transcranial direct current stimulation. The Journal of Physiology, 527, 633–639. DOI: 10.1111/j.1469-7793.2000.t01-1-00633.x 10990547 PMC2270099

[44] Park, D. C., Lautenschlager, G., Hedden, T., Davidson, N. S., Smith, A. D., & Smith, P. K. (2002). Models of visuospatial and verbal memory across the adult life span. Psychology and Aging, 17, 299–320. DOI: 10.1037//0882-7974.17.2.299 12061414

[45] Robison, M. K., McGuirk, W. P., & Unsworth, N. (2017). No evidence for enhancements to visual working memory with transcranial direct current stimulation to prefrontal or posterior parietal cortices. Behavioral Neuroscience, 131(4), 277–280. DOI: 10.1037/bne0000202 28714714

[46] Roe, J. M., Nesheim, M., Mathiesen, N. C., Moberget, T., Alnæs, D., & Sneve, M. H. (2016). The effects of tDCS upon sustained visual attention are dependent on cognitive load. Neuropsychologia, 80, 1–8. DOI: 10.1016/j.neuropsychologia.2015.11.005 26556389

[47] Ruf, S. P., Fallgatter, A. J., & Plewnia, C. (2017). Augmentation of working memory training by transcranial direct current stimulation (tDCS). Scientific Reports, 7, 1–11. DOI: 10.1038/s41598-017-01055-1 28432349 PMC5430723

[48] Simonsohn, U., Nelson, L. D., & Simmons, J. P. (2014). p-curve and effect size: Correcting for publication bias using only significant results. Perspectives on Psychological Science, 9, 666–681. DOI: 10.1177/1745691614553988 26186117

[49] Soveri, A., Lehtonen, M., Karlsson, L. C., Lukasik, K., Antfolk, J., & Laine, M. (2018). Test–retest reliability of five frequently used executive tasks in healthy adults. Applied Neuropsychology: Adult, 25, 155–165. DOI: 10.1080/23279095.2016.1263795 28001452

[50] Sternberg, S. (1966). High-speed scanning in human memory. Science, 153, 652–654. DOI: 10.1126/science.153.3736.652 5939936

[51] Talsma, L. J., Kroese, H. A., & Slagter, H. A. (2017). Boosting cognition: effects of multiple-session transcranial direct current stimulation on working memory. Journal of Cognitive Neuroscience, 29, 755–768. DOI: 10.1162/jocn_a_01077 27897670

[52] Thair, H., Holloway, A. L., Newport, R., & Smith, A. D. (2017). Transcranial direct current stimulation (tDCS): a beginner’s guide for design and implementation. Frontiers in Neuroscience, 11, 641. DOI: 10.3389/fnins.2017.00641 29213226 PMC5702643

[53] Todd, J. J., Fougnie, D., & Marois, R. (2005). Visual short-term memory load suppresses temporo-parietal junction activity and induces inattentional blindness. Psychological Science, 16, 965–972. DOI: 10.1111/j.1467-9280.2005.01645.x 16313661

[54] Todd, J. J., & Marois, R. (2004). Capacity limit of visual short-term memory in human posterior parietal cortex. Nature, 428, 751–754. DOI: 10.1038/nature02466 15085133

[55] Vossel, S., Geng, J. J., & Fink, G. R. (2014). Dorsal and ventral attention systems: distinct neural circuits but collaborative roles. The Neuroscientist, 20, 150–159. DOI: 10.1177/1073858413494269 23835449 PMC4107817

[56] Wang, J., Wen, J. B., & Li, X. L. (2018). No effect of transcranial direct current stimulation of the dorsolateral prefrontal cortex on short-term memory. CNS Neuroscience & Therapeutics, 24, 58–63. DOI: 10.1111/cns.12779 29171169 PMC6489729

[57] Wang, S., Itthipuripat, S., & Ku, Y. (2020). Encoding strategy mediates the effect of electrical stimulation over posterior parietal cortex on visual short-term memory. Cortex, 128, 203–217. DOI: 10.1016/j.cortex.2020.03.005 32361592

[58] Westwood, S. J., & Romani, C. (2018). Null effects on working memory and verbal fluency tasks when applying anodal tDCS to the inferior frontal gyrus of healthy participants. Frontiers in Neuroscience, 12, 166. DOI: 10.3389/fnins.2018.00166 29615855 PMC5867342

[59] Xu, Y., & Chun, M. M. (2006). Dissociable neural mechanisms supporting visual short-term memory for objects. Nature, 440, 91–95. DOI: 10.1038/nature04262 16382240

